# Oral Hygiene Practices, Knowledge, and Awareness of Periodontal Disease Among Pregnant Women Visiting the Antenatal Clinic at Korle‐Bu Teaching Hospital

**DOI:** 10.1002/puh2.70192

**Published:** 2026-02-11

**Authors:** Elijah Kwegyir Johnson, Kwame Adu Okyere Boadu

**Affiliations:** ^1^ University of Ghana Dental School, Korle Bu Accra Ghana; ^2^ Sunyani Teaching Hospital Sunyani Ghana; ^3^ School of Dentistry, College of Health Sciences Kwame Nkrumah University of Science and Technology Kumasi Ghana

**Keywords:** awareness, knowledge, oral hygiene practices, periodontal disease, pregnancy outcomes, pregnant women

## Abstract

**Background:**

Periodontal disease is a chronic inflammatory condition that affects the supporting structures of the teeth. Among pregnant women, hormonal changes further elevate the risk of developing periodontal disease. This study aimed to assess oral hygiene practices, knowledge, and awareness of periodontal disease among pregnant women visiting the antenatal clinic at Korle‐Bu Teaching Hospital (KBTH).

**Methodology:**

A cross‐sectional study was conducted on 122 pregnant women who visited the antenatal clinic at KBTH. The study employed a simple random sampling technique to select participants. A structured questionnaire was used to collect data. Data were analyzed using the Statistical Package for Social Sciences (SPSS) version 26. The significance level was set at *p* < 0.050.

**Results:**

The findings revealed that the majority of participants reported brushing their teeth twice a day (70.5%). However, the rates of flossing, dental visits, and mouthwash usage were low among the participants, with 65.6% reporting never flossing, 69.7% reporting never having a dental visit. The level of knowledge and awareness of periodontal disease was found to be inadequate, with more than half of the participants (54%) demonstrating poor knowledge and awareness. No significant associations were found between oral hygiene practices, demographic characteristics, and knowledge of periodontal disease. However, a significant association was observed between experienced periodontal conditions, such as gum swelling (*p* value = 0.029) and tooth sensitivity (*p* value = 0.013), with knowledge and awareness of periodontal disease.

**Conclusion:**

Although most participants demonstrated good brushing habits, flossing, mouthwash usage, and dental visits were suboptimal. The level of knowledge and awareness of periodontal disease was also found to be limited, with many pregnant women being unaware of its potential impact on pregnancy outcomes. No significant associations were found between oral hygiene practices, demographics, and knowledge of periodontal disease.

## Graphical abstract

This study assessed oral hygiene knowledge, awareness, and practices among pregnant women attending a tertiary antenatal clinic in Ghana, revealing gaps in periodontal disease awareness and suboptimal oral hygiene practices, underscoring the need for integrating oral health education into routine antenatal care services.

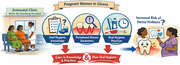

## 1 Introduction

Periodontal disease is a chronic inflammatory condition that affects the supporting structures of the teeth: the gingiva, alveolar bone, cementum, and periodontal ligament [1, 2]. Periodontal disease is a major public health concern, as it is prevalent in populations worldwide and is associated with various systemic diseases, such as diabetes, cardiovascular disease, and preterm birth. Periodontal diseases are caused primarily by infections and inflammation of the gingiva and bone that surround and support the teeth. Periodontal diseases are highly prevalent and can affect up to 90% of the global population [2].

Pregnancy is a critical period for oral health as hormonal changes can increase the risk of periodontal disease. Pregnant women are at an increased risk of developing gingivitis and periodontitis due to hormonal changes that can cause inflammation and bleeding of the gingiva [3, 4, 5]. Hormonal changes during pregnancy can also cause an increase in the volume of blood flowing to the gingiva, which can lead to increased susceptibility to bacterial infection and inflammation [3]. Although periodontal diseases affect up to 90% of the global population [2], the burden is particularly pronounced in West Africa due to varying levels of oral health literacy and access to care. In Ghana, studies indicate that periodontal disease remains a significant public health challenge, yet specific data for the pregnant population at tertiary centers like Korle‐Bu remains limited. Understanding the knowledge, attitudes, and practices in sub‐Saharan Africa is vital, as regional reports often show a gap between high awareness of basic brushing and low utilization of professional preventive services [6, 7]. Women who develop periodontal disease during pregnancy, which is estimated to affect one out of every five, may have a higher risk of adverse pregnancy outcomes [2]. Periodontal disease affects approximately 40% of pregnant women, and the rate is higher among racial and ethnic minorities and low‐income women [8]. Periodontal disease during pregnancy has been linked to a number of adverse outcomes, including preterm birth and low birth weight [9, 10, 11].

A recent systematic review and meta‐analysis focusing on oral health knowledge and practices among pregnant women in sub‐Saharan Africa demonstrated consistently low levels of awareness regarding periodontal disease and its potential impact on pregnancy outcomes [12]. The review further highlighted that most studies originated from East and Southern Africa, with limited data from West African settings, including Ghana. This geographical evidence gap underscores the need for context‐specific studies to generate local data that can inform policy, antenatal health education, and integration of oral healthcare into routine maternal services. Poor oral hygiene and lack of knowledge about periodontal disease can have negative effects on the health of both the mother and fetus. Studies have shown that periodontal disease during pregnancy is associated with an increased risk of preterm birth, low birth weight, and gestational diabetes. A systematic review and meta‐analysis by Polyzos et al. (2009) [13] found that periodontal therapy during pregnancy was associated with a reduction in preterm birth and low birth weight. Similarly, a systematic review and meta‐analysis by Puertas et al. (2018) [14] found that periodontal disease during pregnancy was associated with an increased risk of preterm birth and low birth weight. Good oral hygiene practices during pregnancy have been shown to have a positive impact on pregnancy outcomes. Polyzos et al. [13] discovered that pregnant women who reported good oral hygiene practices were less likely to have preterm births than those who reported poor oral hygiene practices.

Therefore, it is important to assess the oral hygiene practices, knowledge, and awareness of periodontal disease among pregnant women visiting the antenatal clinic at Korle‐Bu Teaching Hospital (KBTH) in order to develop interventions that can improve oral health and reduce the risk of periodontal disease during pregnancy. This is crucial, as poor oral hygiene and lack of knowledge about periodontal disease can have negative effects on the health of both the mother and fetus.

Despite the established association between periodontal disease and adverse pregnancy outcomes, data on oral hygiene practices and periodontal disease awareness among pregnant women in Ghana remain scarce. This study therefore aimed to assess oral hygiene practices, knowledge, and awareness of periodontal disease among pregnant women attending the antenatal clinic at KBTH.

### 1.1 Null Hypothesis

There is no significant association between oral hygiene practices, sociodemographic characteristics, and the level of knowledge and awareness of periodontal disease among pregnant women attending antenatal care at KBTH.

## 2 Methods

### 2.1 Study Area

**FIGURE 1:**
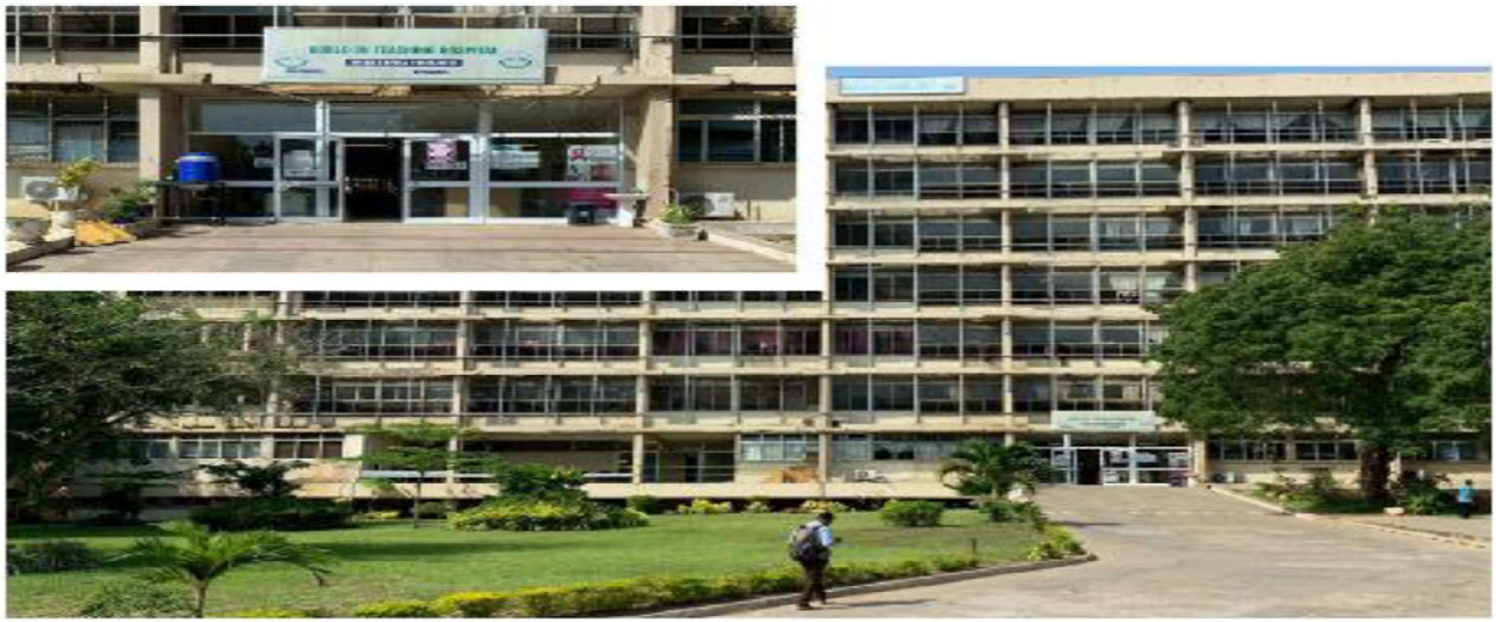
Korle Bu Teaching Hospital.

KBTH was established in 1923, and it is located in the nation's capital Accra, specifically in the Ablekuma South Constituency. It is the largest tertiary hospital in the country and serves as a major referral center for the southern part of Ghana and beyond. It is affiliated with the University of Ghana Medical and Dental Schools and plays a crucial role in medical education and training. It serves as a teaching hospital, providing hands‐on clinical experience for medical and dental students. The KBTH is equipped with multiple departments and centers of medical excellence, offering a wide range of healthcare services (Figure 1).

The Obstetrics and Gynecology department at KBTH is one of the key departments within the hospital. It comprises five units, each with a designated antenatal clinic day. Pregnant women seeking healthcare services at KBTH are typically seen in the gynecology clinic during early pregnancy and are later transferred to the antenatal clinic between 14 and 20 weeks of gestation for ongoing care.

### 2.2 Study Design

A cross‐sectional survey design was utilized to assess oral hygiene practices, knowledge, and awareness of periodontal disease among pregnant women attending the antenatal clinic at KBTH. This cross‐sectional study was conducted and reported in accordance with the Strengthening the Reporting of Observational Studies in Epidemiology (STROBE) guidelines for cross‐sectional studies.

### 2.3 Specific Objectives

1. To assess oral hygiene practices among pregnant women attending the antenatal clinic at KBTH.
2. To determine the level of knowledge and awareness of periodontal disease among pregnant women attending the antenatal clinic.
3. To examine the association between oral hygiene practices and knowledge and awareness of periodontal disease.
4. To assess the relationship between sociodemographic characteristics and knowledge and awareness of periodontal disease among pregnant women.

### 2.4 Study Population

The study population consisted of pregnant women attending the antenatal clinic at KBTH.

### 2.5 Inclusion Criteria

The following inclusion criteria were applied when selecting the participants:

• Pregnant women who provided informed consent to participate in the study.
• Pregnant women attending the antenatal clinic at KBTH during the study period.
• Pregnant women who comprehended and could communicate in English or Twi languages.

### 2.6 Exclusion Criteria

The following exclusion criteria were applied when selecting the participants:

• Pregnant women with medical or obstetric conditions that precluded participation.
• Pregnant women who participated in the pretesting of the questionnaire.

### 2.7 Sampling

A stratified random sampling technique was employed to select participants from the antenatal clinic at KBTH. The clinic's OPD was divided into four sections, labeled A, B, C, and D. To ensure a random selection process, the sections were assigned numbers 1–4 and written on individual pieces of paper. The papers were then placed in a box and thoroughly mixed. One piece of paper from the box was selected without bias or knowledge of the specific sections. This random selection ensured that pregnant women from any of the four sections had an equal chance of being included in the study. The sampling frame comprised all pregnant women attending the antenatal clinic at KBTH during the study period. Daily attendance registers for the selected clinic sections served as the basis for participant selection, ensuring that each eligible pregnant woman had an equal probability of being recruited.

### 2.8 Sample Size Determination

Sample size estimation was performed using the single population proportion formula, appropriate for cross‐sectional studies. A prevalence of 30% knowledge of periodontal disease among pregnant women was assumed on the basis of previous studies in similar settings [15]. Using a 95% confidence level, a margin of error of 5%, and a statistical power of 90%, the minimum required sample size was calculated to be 116 participants. An allowance was made for potential nonresponse; however, a total of 122 pregnant women were successfully recruited.

### 2.9 Data Collection

Data were collected using a self‐administered questionnaire that included both closed and open‐ended questions. The questionnaire was divided into four distinct sections: demographic information, oral hygiene practices, knowledge and awareness of periodontal disease, and knowledge and awareness of periodontal disease on pregnancy outcomes. The questionnaire was pretested on a sample of 10 pregnant women who visited the OPD of the antenatal clinic of KBTH. The pretest was conducted to assess clarity, relevance, and comprehension of questionnaire items, as well as the average time required for completion. Ten participants were considered adequate for pretesting to identify ambiguities and logistical challenges, consistent with methodological recommendations for pilot testing survey instruments. Data from the pretest were excluded from the final analysis.

The questionnaire was adapted from previously published and validated instruments used to assess oral hygiene practices and periodontal disease knowledge among pregnant women [16]. Minor modifications were made to reflect the local context and healthcare setting in Ghana. Content validity was ensured through expert review by dental public health specialists. Data were collected via paper based questionnaires distributed to respondents.

### 2.10 Quality Control

To ensure privacy and confidentiality of the collected data, strict measures were taken. The questionnaires were securely kept by the researcher, and only authorized personnel had access to the data. Participants’ personal information was anonymized and kept confidential, with identifiers removed to maintain privacy. Additionally, a research assistant was recruited and properly trained to assist with data collection, ensuring adherence to research ethics and protocols. To ensure the accuracy and quality of the data, a double‐entry technique was employed, where data entries were independently entered by two individuals and cross‐checked for consistency and accuracy. Checks for erroneous inputs were conducted to identify and correct any errors in data entry, ensuring the integrity and reliability of the collected data.

### 2.11 Data Analysis

The data collected in this study were entered into Microsoft Excel for initial organization and then analyzed using Statistical Package for Social Sciences (SPSS) software (Version 26). Descriptive statistics, such as proportions, frequency tables, and charts, were used to summarize categorical variables. To assess the associations between variables, the chi‐square test of association was employed when appropriate. A significance level of 0.05 was set, and *p* values less than 0.05 were considered statistically significant, indicating a strong association between the variables being analyzed.

### 2.12 Ethical Considerations

Ethical clearance was obtained from the Ethical and Protocol Review Committee of the Department of Community and Preventive Dentistry at the University of Ghana Dental School. An introductory letter was obtained from the University of Ghana Dental School and presented to the head of maternity unit, KBTH, for permission before commencement of data collection. A written consent was obtained from the participants before the commencement of the study. This written consent included an explanation of the purpose and benefits of participating in the study. The participants were made to understand that participation was purely voluntary and that they were free to discontinue at any given time within the study. The participants were given the opportunity to ask questions, and consent was required by way of a signature before administering the questionnaire. Participants were allowed to provide answers to the questionnaire at their own place of convenience to ensure privacy. The questionnaire did not include the names of the participants or any information that could help identify the participants. Data collected were kept under lock and key for use by the principal investigator. Those entered into Excel spreadsheet were kept on a computer with a secure password.

## 3 Results

### 3.1 Sociodemographic Characteristics of Respondents

The age groups of the participants are presented in Table 1. Nearly 60% of the study populations were in the age range of 30–39. Those aged 40 years and above constituted a small fraction (6.6%). More than one‐third of the respondents had tertiary education, followed by SHS and JHS. Three‐quarters of the pregnant women involved in the study were in their third trimester. The remaining quarter were either in their first or second trimester.

**TABLE 1:** Sociodemographic characteristics of respondents (*n* = 122).

Characteristic	Number (%)
Age	
20–29	41 (33.6)
30–39	73 (59.8)
40+	8 (6.6)
Education level	
Primary/JHS	34 (27.9)
Senior high school	40 (32.8)
Tertiary	48 (39.3)
Trimester	
First	5 (4)
Second	26 (21)
Third	91 (75)

*Source:* Survey 2023.

### 3.2 Oral Hygiene Practices

All participants brushed their teeth at least once daily. The majority (70.5%) of the respondents brushed twice daily. Some participants brushed more than twice. Table 2 shows the frequency of brushing. Approximately two‐thirds of the respondents reported never flossing, whereas the majority of the remaining respondents reported irregular flossing habits. Nearly three‐quarters of the participants reported using mouthwashes. However, the majority reported irregular use, whereas a minority of the study participants (27%) reported having never used a mouthwash. Majority of the respondents had never visited the dentist, whereas a few (30%) had visited the dentist in the past. The majority of respondents (86%) had never had professional dental cleaning (scaling and polishing), whereas a little less than one‐quarter of the study participants had undergone professional dental cleaning. Only 2 out of the 122 respondents had a dental checkup during the antenatal period. As displayed in Table 2, a few of the respondents intend on seeing a dentist as part of antenatal care, whereas majority (82%) did not intend on seeing a dentist as part of antenatal.

**TABLE 2:** Oral hygiene practices among respondents (*n* = 122).

Characteristic	Number (%)
Brushing frequency	
Once a day	30 (24.6)
Twice a day	86 (70.5)
More than twice a day	6 (4.9)
Flossing	
Once a day	11 (9)
Twice a day	5 (4)
More than twice a day	1 (1)
Not regularly	24 (20)
Never	81 (66)
Mouthwash use	
Once a day	16 (13)
Twice a day	12 (10)
Not regularly	61 (50)
Never	33 (27)
Routine dental visit	
Yes	37 (30)
No	85 (70)
Professional dental cleaning	
Yes	17 (14)
No	105 (86)
Dental checkup at antenatal	
Yes	2 (2)
No	122 (98)
Intention to see dentist at antenatal	
Yes	22 (18)
No	100 (82)

*Source:* Survey 2023.

## 4 Knowledge and Awareness of Periodontal Diseases

Over two‐thirds of the respondents (68%) were familiar with the term “periodontal/gum disease, ” whereas less than one‐third were not. Nearly one‐third of the participants were aware scaling and polishing help prevents periodontal disease, whereas the majority (68%) were unaware of this. One‐third of the participants were aware that pregnant women were at a higher risk of developing periodontal (gum) disease. In contrast, a large majority (67%) of the respondents were unaware of the increased susceptibility to periodontal disease during pregnancy. Over three‐quarters of the respondents were unaware of a possible link between periodontal disease and pregnancy outcome. The vast majority (89%) of respondents were unaware that periodontal disease may be associated with adverse pregnancy outcomes, such as preterm birth and low birth weight. Only a small fraction (11%) of the participants demonstrated awareness that periodontal disease and adverse pregnancy outcomes may be related. Nearly half of the respondents had adequate knowledge and awareness of periodontal disease, as seen in Table 3.

**TABLE 3:** Knowledge and awareness of periodontal diseases among respondents (*n* = 122).

Characteristic	Number (%)
Heard of periodontal disease	
Yes	83 (68)
No	39 (32)
Awareness of SNP as preventive factor	
Yes	83 (68)
No	39 (32)
Pregnancy as risk factor	
Yes	40 (33)
No	82 (67)
Periodontal disease on pregnancy outcome	
Yes	26 (21)
No	96 (79)
Knowledge and awareness level of periodontal diseases	
Adequate	66 (54)
Poor	56 (46)

*Source:* Survey 2023.

## 5 Reported Knowledge and Experience of the Various Periodontal Conditions Is Presented in Tables 4 and 5

**TABLE 4:** Knowledge of periodontal condition.

Periodontal conditions	Count	Percent
Bleeding gum		
No	26	21.3
Yes	96	78.7
Gum swelling		
No	44	36.1
Yes	78	63.9
Gum overgrowth		
No	112	91.8
Yes	10	8.2
Tooth sensitivity		
No	43	35.2
Yes	79	64.8
Tooth mobility		
No	86	70.5
Yes	36	29.5

*Source:* Survey 2023.

**TABLE 5:** Experience of periodontal condition.

Periodontal condition	Count	Percent
Bleeding gum		
No	82	67.2
Yes	40	32.8
Gum swelling		
No	99	81.1
Yes	23	18.9
Gum overgrowth		
No	122	100.0
Yes	0	0.0
Tooth sensitivity		
No	93	76.2
Yes	29	23.8
Tooth mobility		
No	115	94.3
Yes	7	5.7

*Source:* Survey 2023.

### 5.1 Bleeding Gums

A significant proportion of the participants (78.7%) indicated knowledge of bleeding gums, whereas 21.3% had no knowledge of this condition. About one‐third of the participants reported having experienced bleeding gums, compared to two‐thirds who did not have this symptom.

### 5.2 Gum Swelling

More than half of the participants indicated knowledge of gum swelling, whereas a little above one‐third were unaware of this condition. In terms of experience, less than one‐fifth reported having gum swelling, whereas the majority (over four‐fifths) did not report this symptom.

### 5.3 Gum Overgrowth

Only a small fraction (approximately 1–10th) of participants indicated knowledge of gum overgrowth. The vast majority (over 9–10ths) did not know about this symptom. None of the participants (0%) reported experiencing gum overgrowth.

### 5.4 Tooth Sensitivity

Approximately two‐thirds of the participants indicated some knowledge of tooth sensitivity, whereas around one‐third were unaware. With regard to experience, approximately one‐quarter reported having tooth sensitivity compared to three‐quarters who did not.

### 5.5 Tooth Mobility

Only about one‐third of participants indicated knowledge of tooth mobility, with the majority (around two‐thirds) being unaware. In terms of experience, only a small fraction (approximately 1–20th) reported having tooth mobility, whereas the majority (over 95%) did not have this symptom.

## 6 Reported Experience of Periodontal Condition During Current Pregnancy Is Depicted in Table 6

**TABLE 6:** Experience of periodontal condition during current pregnancy.

Periodontal condition	Count	Percent
Bleeding gum		
No	110	90.2
Yes	12	9.8
Gum swelling		
No	115	94.3
Yes	7	5.7
Gum overgrowth		
No	122	100.0
Yes	0	0.0
Tooth sensitivity		
No	117	95.9
Yes	5	4.1
Tooth mobility		
No	121	99.2
Yes	1	0.8

*Source:* Survey 2023.

### 6.1 Bleeding Gums

Only a small minority (9.8%) of participants reported experiencing bleeding gums during their current pregnancy, whereas the vast majority (90.2%) did not experience this symptom.

### 6.2 Gum Swelling

An even smaller fraction (5.7%) of participants experienced gum swelling during their current pregnancy, with the overwhelming majority (94.3%) not reporting this symptom.

### 6.3 Gum Overgrowth

None of the participants (0%) reported experiencing gum overgrowth during their current pregnancy, indicating that this condition was absent in the entire sample.

### 6.4 Tooth Sensitivity

The vast majority of the participants (95.9%) did not experience tooth sensitivity during pregnancy.

### 6.5 Tooth Mobility

The data show that tooth mobility was the least frequently reported condition, with only 0.8% of the participants experiencing it during pregnancy.

## 7 Association Between Knowledge and Awareness Level of Periodontal Disease as Against Oral Hygiene Practices, Sociodemographics and Reported Periodontal Disease During Pregnancy

There was no significant association between knowledge and awareness level of periodontal disease and the various oral hygiene practices, as depicted in Table 7.

**TABLE 7:** Association between knowledge and awareness levels and oral hygiene practices.

	Knowledge and awareness level			
Oral hygiene practice	Poor count	Adequate count	Total	*p* value
Frequency of brushing				0.216
Once	18	12	30	
Twice	43	43	86	
More than 2×	5	1	6	
Total	66	56	122	
Frequency of flossing				0.376
Once	9	2	11	
Twice	3	2	5	
More than 2×	1	1	2	
Not regularly	11	13	24	
Never	42	38	80	
Total	66	56	122	
Mouthwash usage				0.816
Once	9	7	16	
Twice	6	6	12	
Not regularly	31	30	61	
Never	20	13	33	
Total	66	56	122	
Dental visit				0.686
Yes	19	18	37	
No	47	38	85	
Total	66	56	122	
Dental scaling				0.530
Yes	8	9	17	
No	58	47	105	
Total	66	56	122	

*Source:* Survey 2023.

### 7.1 Association Between Knowledge and Awareness Level of Periodontal Diseases and Sociodemographic Features

No significant association was found between the sociodemographic characteristics and knowledge and awareness level of periodontal disease, as shown in Table 8.

**TABLE 8:** Association between knowledge and awareness level and reported periodontal condition.

	Knowledge and awareness level			
Characteristics	Poor count	Adequate count	Total	*p* value
Age group				0.602
20–29	22	19	41	
30–39	41	32	73	
40+	3	5	8	
Total	66	56	122	
Occupation				0.479
Employed	24	21	45	
Self‐employed	38	30	68	
Student	0	2	2	
Unemployed	4	3	7	
Total	66	56	122	
Educational level				0.076
JHS	24	10	34	
SHS	19	21	40	
Tertiary	23	25	48	
Total	66	56	122	
Trimester				0.873
First	3	2	5	
Second	13	13	26	
Third	50	41	91	
Total	66	56	122	

*Source:* Survey 2023.

The level of knowledge and awareness of periodontal disease was significantly associated with two of the experienced periodontal conditions: gum swelling (*p* value = 0.029) and tooth sensitivity (*p* value = 0.013) (Table 9).

**TABLE 9:** Association between knowledge and awareness level and reported periodontal condition during pregnancy.

	Knowledge and awareness level			
Periodontal condition	Poor count	Adequate count	Total	*p* value
Bleeding gums				0.764
Yes	6	6	12	
No	60	50	110	
Total	66	56	122	
Gum swelling				0.029*
Yes	1	6	7	
No	65	50	115	
Total	66	56	122	
Tooth sensitivity				0.013*
Yes	0	5	5	
No	66	51	51	
Total	66	56	122	
Tooth mobility				0.276
Yes	0	1	1	
No	66	55	121	
Total	66	56	122	

*Source:* Survey 2023.

* Significant at *p* < 0.05.

## 8 Discussion

### 8.1 Demographic Characteristics

A total of 122 pregnant women were involved in the study. In terms of age range, the majority of participants were in the 30–39 age range (59.8%), followed by the 20–29 age range (33.6%). A small proportion of participants were aged 40 years and above (6.6%). The mean age of the respondents with a standard deviation was 31.57 ± 5.19 years. All participants included in the study had received some level of formal education, with a notable proportion (39.3%) having attained tertiary education. This finding is consistent with a study conducted by Togoo et al. (2019) [16], who reported that the majority of pregnant women (53.6%) had a tertiary education. Furthermore, 32.8% of the participants in the study had completed secondary education (SHS), whereas 27.9% had received some form of basic education (primary/JHS). The majority of the study participants were in their third trimester (74.6%), followed by the second trimester (21.3%), and a small proportion were in their first trimester (4.1%).

### 8.2 Oral Hygiene Practices

Oral hygiene practices of the study participants were assessed using a structured questionnaire. The questionnaire included questions related to brushing frequency, flossing habits, mouthwash use, and dental visit patterns. Maintaining good oral hygiene practices, such as regular brushing, is crucial in preventing dental plaque buildup and reducing the risk of gingivitis, especially during pregnancy when hormonal changes increase the susceptibility to gingival disease [17, 18]. It is encouraging to note that a significant proportion of pregnant women in this study (70.5%) reported brushing their teeth twice a day, in line with recommendations from dental professionals and health organizations [19, 20]. This finding aligns with previous research conducted by both Hullah and Okyere Boadu et al. [21, 22] and which also reported high frequencies of twice‐daily brushing among pregnant women.

Furthermore, it is important to highlight that all 122 (100%) respondents in the study reported using toothpaste and a toothbrush for brushing, indicating a widespread awareness of the importance of these oral hygiene tools. This reflects a positive trend in brushing habits among the pregnant women visiting the antenatal clinic. A small proportion of participants (24.6%) reported brushing their teeth once a day, whereas a very small percentage (4.9%) reported brushing more than twice a day.

Flossing plays a crucial role in removing plaque and debris from the interdental spaces, which cannot be achieved by brushing alone [23]. In terms of flossing frequency, the findings revealed a concerning trend, with the majority of participants reporting never flossing (65.6%) or not flossing regularly (19.7%). These findings are consistent with those of Okyere Boadu et al. [22] and Togoo et al. [16], who identified low flossing rates among pregnant women. However, the percentage of pregnant women who had never flossed was lower (48.3) in a study conducted by Chinenye‐Julius et al. [24].

There was low a utilization of mouthwash among the pregnant women (13.1% reported usage once a day and 9.8% twice a day), indicating a potential lack of awareness regarding the benefits of mouthwash in oral hygiene practices. Mouthwash can reduce bacterial load and improve oral health [25], especially during pregnancy, when hormonal changes may exacerbate gingival inflammation. A cross‐sectional study in India reported that only 16.5% of pregnant women used mouthwash as part of their oral hygiene practices and that mouthwash users had better oral health status than nonusers [26].

Regarding dental visits, a considerable proportion of participants (69.7%) reported never visiting the dentist, whereas 30.3% reported ever visiting the dentist. These findings are consistent with those of previous studies that have highlighted suboptimal dental attendance among pregnant women [27, 28]. On the contrary, a study conducted by Gao et al. (2021) [29] among pregnant women in Australia showed that the majority of the respondents (96.9%) had visited a dentist before. This may be due to an increased assess to dental services.

It was observed that the majority of respondents did not intend to see a dentist as part of antenatal care. Regular dental visits during pregnancy are crucial for preventive dental care, the early detection of oral health issues, and necessary treatments. The low rate of dental visits observed in this study population indicates a potential barrier to accessing oral healthcare services during pregnancy. It is essential to address barriers, such as financial constraints, lack of awareness, and misconceptions about dental care during pregnancy, to encourage regular dental visits among pregnant women.

### 8.3 Knowledge and Awareness of Periodontal Disease Among Participants

The level of knowledge and awareness regarding periodontal disease among the study participants was assessed by summing the responses to questions related to their knowledge and experience of periodontal disease. A total score of 12 was assigned, with participants scoring below 6 considered to have poor knowledge and awareness and those scoring above 6 deemed to have adequate knowledge and awareness.

The findings revealed that slightly more than half of the participants (54%) had poor knowledge and awareness of periodontal disease, whereas the remaining 46% demonstrated adequate knowledge and awareness. These results are consistent with previous studies conducted among pregnant women, indicating limited or poor knowledge and awareness of periodontal disease [30, 31].

The level of knowledge and awareness of periodontal disease was not significantly associated with any of the participants’ oral hygiene practices or sociodemographic characteristics. This suggests that other factors, such as personal beliefs, attitudes, motivation, and perceived barriers, may influence the oral hygiene behavior and practices of pregnant women [32, 33]. Therefore, it is important to explore these factors in future studies and to design appropriate strategies to improve the oral hygiene practices of pregnant women.

The level of knowledge and awareness of periodontal disease was significantly associated with two of the experienced periodontal conditions: gum swelling (*p* value = 0.029) and tooth sensitivity (*p* value = 0.013). This indicates that some pregnant women may have recognized the signs and symptoms of periodontal disease and sought information or treatment for their condition. However, no significant association was found between the level of knowledge and awareness of periodontal disease and the other experienced periodontal conditions: bleeding gum, tooth mobility, and gum overgrowth. This may imply that some pregnant women may have ignored or underestimated these conditions or attributed them to normal changes during pregnancy. Therefore, it is essential to educate pregnant women about the potential causes and consequences of these conditions and to encourage them to seek regular dental care during pregnancy.

### 8.4 Knowledge and Awareness of Periodontal Disease on Pregnancy Outcomes

The knowledge and awareness of the potential link between periodontal disease and pregnancy outcomes were assessed among the study participants. The findings revealed that a significant proportion of the respondents, approximately 79%, were unaware of the possible association between periodontal disease and pregnancy outcomes. This lack of awareness is concerning, as periodontal disease has been implicated in adverse pregnancy outcomes, such as low birth weight and preterm birth [34, 35, 36].

These findings align with previous research studies. A study by Ratre et al. [37] conducted among pregnant women found that a large majority of the participants had limited knowledge and awareness of the impact of periodontal disease on pregnancy outcomes. Similarly, another study by Ramamurthy et al. [31] reported a general lack of awareness among pregnant women regarding the potential risks of periodontal disease on adverse pregnancy outcomes.

Furthermore, the present study found that only a small percentage of the respondents, specifically 11, were aware that periodontal disease may lead to low birth weight and preterm birth. This finding is consistent with that of a study by Nwankwo et al. [7] that evaluated the knowledge of the relationship between periodontal disease and adverse pregnancy outcomes among 200 pregnant women in Nigeria. The study found that the participants had little knowledge of the relationship between periodontal disease and adverse pregnancy outcomes and that only 13% of them were aware of the possible link between periodontal disease and preterm birth or low birth weight.

The limited awareness among pregnant women regarding the potential risks of periodontal disease on pregnancy outcomes is a cause for concern. This highlights the need for comprehensive education and awareness campaigns targeting pregnant women, healthcare providers, and the general public. Effective communication strategies, such as the integration of oral health education into routine prenatal care visits and dissemination of informational materials, can play a crucial role in improving knowledge and awareness.

## 9 Limitations

This study has several limitations. The cross‐sectional design limits causal inference between oral hygiene practices and periodontal disease knowledge. Data were self‐reported and may be subject to recall and social desirability bias. Additionally, the single‐center setting may limit the generalizability of findings to other regions of Ghana. Income level, religious affiliation, and parity were not assessed in this study due to time constraints and concerns regarding participant burden during antenatal clinic visits. However, these variables are known to influence health‐seeking behavior and oral health practices and should be considered in future studies to provide a more comprehensive understanding of determinants of periodontal health among pregnant women. Despite these limitations, the study provides valuable baseline data to inform maternal oral health interventions.

## 10 Recommendations

On the basis of the findings of this study, several key recommendations emerge to improve oral hygiene practices, knowledge, and awareness of periodontal disease among pregnant women visiting the antenatal clinic at KBTH:

Develop targeted oral health education programs within antenatal care settings: It is crucial to implement educational programs that specifically address the unique oral health needs of pregnant women. These programs should focus on promoting good oral hygiene practices, such as regular brushing, flossing, and mouthwash use, while highlighting the potential impact of periodontal disease on pregnancy outcomes. Tailoring the information to the local context and language barriers will enhance the effectiveness of these programs.

Foster collaboration and communication between healthcare providers: Encourage multidisciplinary collaboration between dental professionals, obstetricians, midwives, and other healthcare providers involved in prenatal care. This collaboration should involve regular knowledge‐sharing sessions, joint training programs, and integrated care approaches. By working together, healthcare professionals can provide comprehensive oral healthcare and education to pregnant women, ensuring continuity of care throughout pregnancy.

Incorporate routine oral health screenings into prenatal care: Integrate oral health screenings as a standard component of prenatal care visits. This will allow for early detection and management of periodontal diseases. Dental professionals should be involved in conducting these screenings, providing appropriate treatment and referrals, and delivering oral health education tailored to the individual needs of each pregnant woman.

Advocate for policy changes and guidelines: There should be work toward the development and implementation of policies and guidelines that prioritize oral healthcare during pregnancy. This includes advocating for reimbursement for dental services related to prenatal oral health and promoting the inclusion of oral health indicators in national maternal and child health programs. By establishing these policies, governments and healthcare systems can emphasize the importance of oral health as an integral part of prenatal care.

Future research: Encourage further research to explore the long‐term effects of improved oral hygiene practices and increased knowledge of periodontal disease on pregnancy outcomes. Additionally, investigations into the effectiveness of different educational interventions and strategies to promote oral health during pregnancy would be valuable.

## 11 Conclusion

In conclusion, this study focused on examining oral hygiene practices, knowledge, and awareness of periodontal disease among pregnant women visiting the antenatal clinic at KBTH. The findings of this study shed light on the existing gaps and areas for improvement in oral healthcare during pregnancy.

The results revealed that the majority of participants demonstrated commendable brushing habits by engaging in twice‐daily brushing with toothpaste and toothbrush. Adherence to regular brushing is a positive indicator of commitment to oral health maintenance during pregnancy. However, it is essential to address the suboptimal level of other oral hygiene practices observed in this study, such as flossing, mouthwash usage, and regular dental visits. These areas present opportunities for targeted interventions and education to improve overall oral hygiene behaviors among pregnant women.

Additionally, the study revealed a limited level of knowledge and awareness of periodontal disease among the participants. A concerning lack of awareness among the majority of pregnant women regarding the potential impact of periodontal disease on pregnancy outcomes was observed. This highlights the need for increased education and awareness campaigns to inform pregnant women about the importance of oral health during pregnancy and its potential impact on both maternal and infant health.

Finally, the study identified no significant associations between oral hygiene practices, demographic characteristics, and knowledge and awareness of periodontal disease. However, experienced periodontal conditions, such as gum swelling and tooth sensitivity, were found to be associated with knowledge and awareness of periodontal disease.

## Author Contributions

Elijah Kwegyir Johnson and Kwame Adu Okyere Boadu contributed to the concept, visualization, methodology, design, data analysis, and manuscript write‐ups. All authors have read and approved the final version of the manuscript. Kwame Adu Okyere Boadu had full access to all of the data in this study and takes complete responsibility for the integrity of the data and the accuracy of the data analysis.

## Funding

The authors have nothing to report.

## Conflicts of Interest

The authors declare no conflicts of interest.

## Transparency Statement

Kwame Adu Okyere Boadu affirms that this manuscript is an honest, accurate, and transparent account of the study being reported; that no important aspects of the study have been omitted; and that any discrepancies from the study as planned have been explained.

## Data Availability

The authors confirm that the data supporting the findings of this study are available within the article.
